# Dual targeting ovarian cancer by Muc16 CAR-T cells secreting a bispecific T cell engager antibody for an intracellular tumor antigen WT1

**DOI:** 10.21203/rs.3.rs-2887299/v1

**Published:** 2023-05-08

**Authors:** Sung Soo Mun, Leila Peraro, Jeremy Meyerberg, Tatyana Korontsvit, Manish Malviya, Thomas Gardner, Chrisann Kyi, Roisin E. O’Cearbhaill, Cheng Liu, Tao Dao, David A. Scheinberg

**Affiliations:** Memorial Sloan Kettering Cancer Center; Memorial Sloan Kettering Cancer Center; Memorial Sloan Kettering Cancer Center; Memorial Sloan Kettering Cancer Center; Memorial Sloan Kettering Cancer Center; Memorial Sloan Kettering Cancer Center; Memorial Sloan Kettering Cancer Center; Memorial Sloan Kettering Cancer Center; Eureka Therapeutics, Inc; Memorial Sloan Kettering Cancer Center; Memorial Sloan Kettering Cancer Center

## Abstract

Epithelial ovarian cancer is the most lethal of gynecological cancers. The therapeutic efficacy of chimeric antigen receptor (CAR) T cell directed against single antigens is limited by the heterogeneous target antigen expression in epithelial ovarian tumors. To overcome this limitation, we describe an engineered cell with both dual targeting and orthogonal cytotoxic modalities directed against two tumor antigens that are highly expressed on ovarian cancer cells: cell surface Muc16 and intracellular WT1. Muc16-specific CAR-T cells (4H11) were engineered to secrete a bispecific T cell engager (BiTE) constructed from a TCR mimic antibody (ESK1) reactive with the WT1-derived epitope RMFPNAPYL (RMF) presented by HLA-A2 molecules. The secreted ESK1 BiTE recruited and redirected other T cells to WT1 on the tumor cells. We show that ESK1 BiTE-secreting 4H11 CAR-T cells exhibited enhanced anticancer activity against cancer cells with low Muc16 expression, compared to 4H11 CAR-T cells alone, both in vitro and in mouse tumor models. Dual orthogonal cytotoxic modalities with different specificities targeting both surface and intracellular tumor-associated antigens present a promising strategy to overcome resistance to CAR-T cell therapy in epithelial ovarian cancer and other cancers.

## Introduction

Epithelial ovarian cancer (EOC) is the most lethal gynecologic malignancy with five-year survival below 30% (1, 2). Despite aggressive treatment regimens, consisting of surgical tumor debulking complemented by platinum-based chemotherapy (3), sometimes in combination with bevacizumab and/or PARP inhibitor maintenance (4, 5), the majority of patients with advanced ovarian cancer relapse; hence there is a strong unmet need for a therapeutic strategy that can produce durable responses and extend overall survival. Although significant clinical advances have been achieved in recent years within the field of cancer immunotherapy, there is no formal FDA approval for immunotherapy in EOC, apart from pembrolizumab in the very rare case of tumor mutational burden-high disease (5).

Targeted immunotherapies using adoptive cell transfer of engineered autologous chimeric antigen receptor (CAR) T cells have shown remarkable clinical results in hematologic malignancies (6-8). In contrast, CAR T-cell approaches against solid tumors have so far only led to infrequent responses. Impaired CAR T-cell proliferation and persistence *in vivo*, the immunosuppressive tumor microenvironment, limited CAR T-cell trafficking to the tumor, and long-term exhaustion of CAR T-cell functions all have proven to be key obstacles for the success of CAR T-cell therapy (9). In addition to these common problems in CART therapy, EOC, in particular, is extremely heterogenous, encompassing different entities with distinct clinicopathological features, genomic profiling and hence, diverse antigen expression (1, 2, 10). Therefore, CAR-T cell therapy targeting single antigens in EOC could be limited, due to the heterogeneous target antigen expression and outgrowth of tumors lacking the antigen targeted by CAR-T cells. Some of the common cell surface antigens (mesothelin, Muc16, HER2, and folate-receptors) that are overexpressed on ovarian cancer cells have been investigated in CAR-T cell development (11). A CAR-T cell trial against mesothelin in 15 advanced solid tumors, including patients with EOC, showed that the treatment was well tolerated, but with only stable disease (SD) as the best overall response (12). Muc16 (CA125) CAR T cells, modified to secrete IL-12, were tested in a phase I trial using i.v. and i.p. administration with “stable disease” as the best observed clinical response in a cohort of 18 heavily pretreated patients with EOC (4, 13). These results suggested that CART cell therapy for EOC faces an urgent challenge to find EOC-selective targets, in order to improve the efficacy of EOC CART cells.

Spontaneous anti-tumor responses with cytotoxic T lymphocytes have been detected in patients, and the presence of tumor infiltrating lymphocytes (TILs) is correlated with a better prognosis and improved survival in patients with EOC(14) (15,16). These studies clearly illustrated the effective T cell responses directed against intracellular tumor antigens, which, compared to surface lineage antigens, account for the most tumor-specific antigens, compared to surface lineage antigens (17). T cell receptor mimic mAbs (TCRm) that recognize peptide/MHC complex are emerging as potential candidates for directing CAR T cells to these antigens. Wilm’s tumor (WT)1 is an intracellular, oncogenic transcription factor that is overexpressed in a wide range of leukemias and solid cancers including EOC. RMFPNAPYL (RMF), a WT1-derived CD8 + T cell human leukocyte antigen (HLA)–A0201 epitope, is a validated target for T cell–based immunotherapy in numerous clinical trials (18-21). We are exploring the use of a WT1-specific vaccine in EOC currently(22). In addition, we previously described the generation and therapeutic efficacy of a human TCRm mAb, ESK1, specific for the WT1-derived RMF epitope in the context of HLA-A*02:01 molecule. The TCRm was also engineered into BiTE and CAR T cell formats that showed effective killing of ovarian cancer cell lines in animal models (23, 24). Importantly, ESK1-BiTE also induced epitope spreading to other EOC antigen such as a Her2neu-derived epitope (23), suggesting that this BiTE was able to reactivate pre-existing anti-tumor T cell responses, conveying the potential to broadening the anti-tumor immune responses.

Here we propose to overcome antigenic heterogeneity of EOC and to enhance local antitumor activity in EOC, by engineering the well-studied CAR T cells specific for Muc16 (4H11) to also secrete the ESK1-BiTE against WT1 RMF/HLA-A2 complex, thereby generating dual specificities, as well as orthogonal modalities of killing. We demonstrated that this approach could mediate potent and specific antitumor activity against EOC cells, thereby potentially mitigating the effects of antigen heterogeneity and low expression of Muc16 in EOC.

## Materials and Methods

### Cell samples, cell lines and antibodies.

After informed consent on Memorial Sloan-Kettering Cancer Center Institutional Review Board approved protocols, peripheral blood mononuclear cells (PBMCs) from HLA-typed healthy donors and patients were obtained by Ficoll density centrifugation. The cell lines used in this study were obtained from ATCC (Manassas, VA). The cell lines include: Ovarian cancer cell line SKVO-3, AML cell lines AML-14 and HL-60, T leukemia cell line Jurkat. All tumor cells were HLA typed. Primary ovarian cancer cells were obtained from ascitic fluid, blood and tissue samples from patients with ovarian cancer, following Institutional Review Board (IRB) approval. patients with ovarian cancer. The cell lines were cultured in RPMI 1640 supplemented with 5% FCS, penicillin, streptomycin, 2 mmol/L glutamine, and 2-mercaptoethanol at 37 C/5% CO_2_. Cells were checked regularly for mycoplasma. SKOV-3 cell line was transduced with HLA-A*02:01 molecule as described by Latouche (25), using HLA-A*02:01 SFG vector that was a gift from Dr. Michell Sadelain (MSKCC, New York). SKOV-3 cell line overexpressing Muc16 antigen, SKOV3-MUC16^ectO^ was a gift from Dr. Renier Brentjens, at MSKCC. Tumor cells for all animal studies were transduced with GFP/luciferase as described previously (23). ESK1.BiTE and its control, human or mouse ESK1 and its control human IgG1 or mouse IgG1 were produced by Eureka Therapeutics Inc. (Emeryville, CA), and APC conjugation was done according to the instructions of the manufacturer (Abcam, ab201807). Mab against Muc16 (clone 4H11) was a kind gift from Dr. Renier Brentjens. Mab against human HLA A*02 (clone BB7.2), its isotype control mouse IgG2b (clone MPC-11), human CD3 (clone HIT3A or OKT3), conjugated to various fluorophores, human CD45 mAb (HI30), mouse anti-His tag mAb (clone F24-796) conjugated to FITC or PE, were purchased from BD Biosciences, (San Diego, CA). Recombinant anti-WT1 protein mAb (CAN-R9(IHC)-56-2) was purchased from Abcam (USA).

## Flow cytometry analysis

Muc16 expression on ovarian cancer cell line SKOV-3 was measured by staining the tumor cells with 4H11 mAb conjugated to APC. SKOV-3/HLA-A2 + cell lines were stained with ESK1 mouse IgG1 conjugated to APC, which recognizes WT1-drived RMF epitope in the context of HLA-A0201. For binding by BiTEs, human T cells or cancer cells were incubated with different concentrations of ESK1-BiTE or control BiTE for 30 min on ice, washed, and incubated with secondary mAbs against His-Tag. In some cases, direct staining was performed by using ESK1-BiTE or its control conjugated to APC. HLA-A*02 expression was determined by direct staining of the cells with mAb clone BB7. WT1 intracellular staining was performed by staining the cells with mAb to WT1 (CAN-R9) and its IgG isotype both conjugated to Alexa488 using intracellular fixation and permeabilization buffer set (eBioscience, 88-8824-00). WT1RMF/HLA-A2 expression in Primary ovarian cancer cells were measured by staining the cells with anti-human CD45 mAb vs mouse ESK1. Flow cytometry data were collected on a LSR Fortessa (BD) and analyzed with FlowJo V10.

## Viral constructs

Retroviral vectors were generated using the SFG backbone as previously shown (26, 27). We constructed vectors encoding second-generation CARs targeted to the Muc16 ectodomain or CD19 antigen, with 4-1BB and CD3ζ signaling domains. For the BiTE-secreting CAR T cells, the ESK1 bite sequence was incorporated into the construct separated by a 2A self-cleaving peptide sequence. Retroviral transduction of primary T cells was performed using engineered HEK293T, known as Galv9, which are stable retroviral producer lines that has been previously described (27).

### CAR-T-cell production and characterization.

Human T cells were purified from healthy donor under an Institutional Review Board-exempt protocol. In brief, bulk human T cells were activated on day 0 using CD3/CD28 Dynabeads (Miltenyi) and cultured in RPMI 1640 medium supplemented with 10% FBS and 100 IU recombinant human IL-2. Lentiviral transduction of cells was performed on day 2 and day 3 by centrifugation of activated T cells in media containing virus from retroviral producers Galv-9 cells at 2000xg at room temperature for two hours on RetroNectin-coated plates (Takara Bio, T100A/B). Transduction efficiency was determined by flow cytometry using a Alexa 647-labeled anti-idiotype antibody against the Muc16-targeted CAR (mAb clone 22G6), generated at MSKCC Antibody and Bioresource Core Facility).

### Detection of ESK1-BiTE secretion.

Secretion of the ESK1 BiTE was confirmed by analyzing conditioned media of Galv9 producer cells by Western blot as previously shown (28). Ni-NTA beads were used to ‘pull down’ ESK1 bite from the medium supernatants of WT, 4H11BBζ, and ESK1-4H11BBζ Galv9 cells. Protein was separated using SDS-PAGE and analyzed by immunoblotting using an anti-His-tag antibody. The ESK1 bite appeared at the predicted molecular weight (∽58 kDa) in the conditioned media of ESK1-4H11BBζ Galv9s.

### ESK1 BiTE secretion by CAR T cells and functional assays.

Supernatants of CAR T cells transduced with the CAR/BiTE construct was collected on day 5 and 7, pooled, concentrated 30–40 folds by centrifugation using Amicon Centrifugal Filter Units (Milipore Sigma, UFC801024) at 4000xg for 20 minutes in room temperature. Supernatant fluids were stored at 4°C, and the secretion of ESK1-BiTE was measured by staining SKOV3 and Jurkat cells, and flow cytometric analysis. Recombinant ESK1-BiTE was used in parallel to determine the approximate concentrations of the secreted BiTE. Functional assay of ESK1-BiTE was measured by cytolytic activity of the supernatants by using isolated PBMC’s from the same donor as effector cells. In brief, supernatant fluids in serial dilution were incubated with target cells and isolated PBMC’s at E:T ratio of 10:1, and the cytotoxicity was measured by luciferase-based assay.

### Cytotoxicity assays.

For ESK1-BiTE-mediated cytotoxicity, PBMCs from healthy donors were incubated with targets at a E: T ratio of 20 or 30:1, in the presence or absence of ESK1 or control BiTEs at various concentration for 5 hr. The cytotoxicity was measured by standard ^51^Cr-release assay. For measuring cytotoxicity of CAR T cells, T cells were incubated with luciferase-expressing tumor targets at various E: T ratios for 24 hours. Next day, additional Mock-T cells from the same donor were added for another 24 hours. Remaining luciferase activity was subsequently measured with a Tecan infinite M1000 Pro Microplate Reader. Percentage specific lysis was calculated by the following equation: Percentage = [(target cells alone relative luminescence units (RLU) – total RLU)/(target cells only RLU)] × 100.

### Animal models.

Seven to 8-week-old male NOD.Cg-*Prkdc* SCID IL2*rgtm1*Wjl/SzJ mice, known as NSG, were purchased from the Jackson Laboratory, and were used for xenograft animal tumor model. SKOV3 ovarian cancer cells transduced with HLA-A2 and GFP/luciferase were used. For ESK1 BiTE therapy, 2.5 million Muc16 low SKOV3/A2+/GFP + cells were ip injected into mice. On day3, after confirming the tumor engraftment by bioluminescence imaging (BLI), PBMCs (10 million/mouse) from a healthy donor were injected ip into mice.

A few hours later, ESK or control BiTE was ip injected into mice (5ug/mouse) and repeated on a daily basis. The tumor growth was monitored by BLI after 4 and 9 injections. For 4H11 CART cell therapy, the same Muc16 low SKOV3/A2 + ovarian cancer cells (2 million/mouse) were injected into the peritoneal cavity (ip) of mice and the tumor engraftment was confirmed on day 4 by bioluminescence imaging and mice were randomized into treatment groups. Two million of CAR T cells were ip. injected into mice, followed by 2 million Mock-T cell injection the next day. Tumor growth was monitored by firefly luciferase imaging once a week, for all the animal models. The P test used for the survival curve is Mantel-Cox.

## Results

### Therapeutic activity of ESK1-BiTE in ovarian cancer

**Figure 1:**
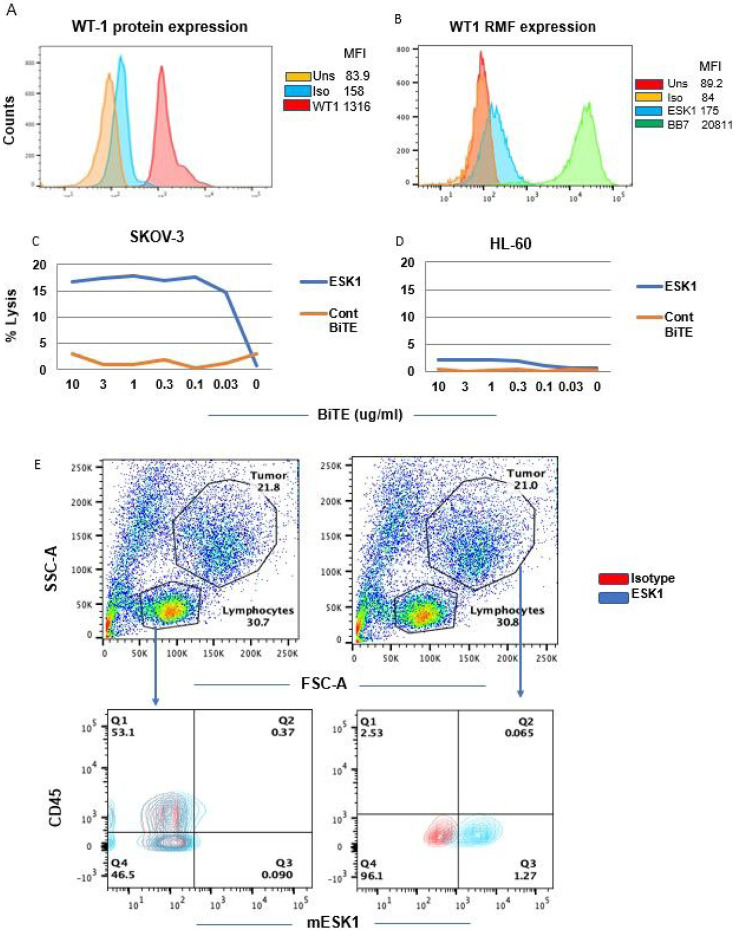
Cytolytic activity of ESK1-BiTE against ovarian cancer cells. (A.) Expression of WT1 protein was measured by intracellular staining of the SKOV3 Muc16 low cells using mAb to WT1 protein. (B). Expression of the cell surface WT1 epitope RMF/HLA-A2 complex was measured by staining the cells with mouse ESK1 mAb or its control at 3ug/ml and analyzed by flow cytometry. (C and D) ESK1.BiTE-mediated T cell cytotoxicity against SKOV3/A2 (C) or HL-60 (D). PBMCs from a healthy donor were incubated with SKOV3 Muc16 low target cells at a E:T ratio of 30:1 in the presence or absence of BiTEs at the indicated concentrations for 5 hr. The BiTE-mediated T cell cytotoxicity was measured by standard ^51^Cr-release assay. The HL-60 AML cell line (HLA-A2−) was used as a control. (E) Binding of ESK1 to fresh tumor cells in ascites from an ovarian cancer patient. Ascites cells were stained with mouse ESK1 vs anti-CD45 or its isotype control, to distinguish lymphocytes from tumor cells. Smaller lymphocytes and large tumor cells were gated and the CD45 vs ESK1 staining was shown in each gate (lower panels).

**Figure 2:**
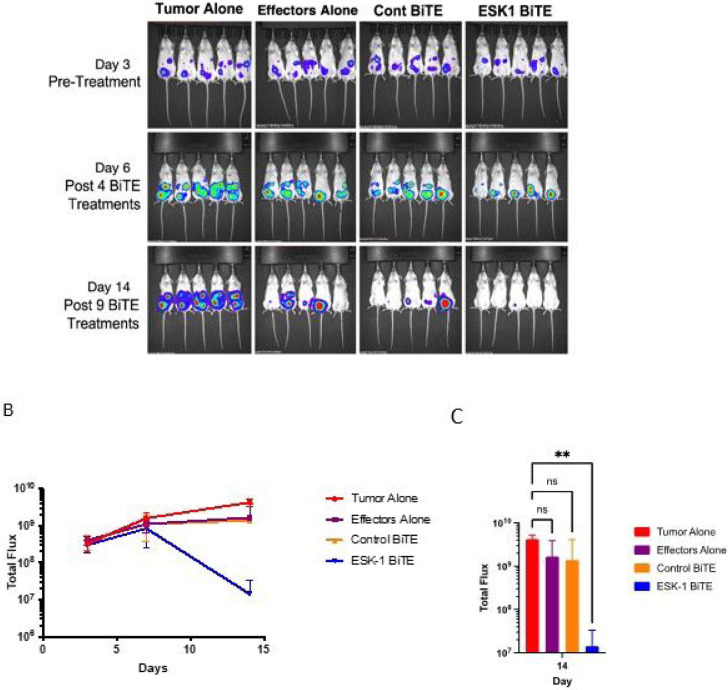
Therapeutic activity of ESK1-BiTE against ovarian cancer cells in vivo. SKOV3 Muc16low (2.5 million cells) were ip injected into NSG mice and tumor engraftment was confirmed on day 3 by bioluminescent imaging. PBMCs (10 million/mouse) from a healthy donor were injected ip into mice. A few hours later, ESK1 or control BiTE was injected ip into mice (5ug/mouse) and the BiTE dosing was repeated in a daily basis for total nine days. Tumor growth was monitored by BLI after 4 and 9 injections (A). Tumor burden was calculated by summing the luminescent signal of each mouse and average signal for each group (*n* = 5 per group) plus/minus SD is plotted (B). Total flux on day 14 post-therapy was shown for each experimental group and *p* Value is determined by 2 way ANOVA test (C).

Expression of the WT1 protein and the WT1 RMF epitope in ovarian cancer cells SKOV3/HLA-A2 + was shown by intracellular staining and by binding of ESK1 to the RMF/HLA-A2 complex (Fig. 1A and B). Consistent with the binding, ESK1-BiTE also redirected T cell cytotoxicity against SKOV3/HLA-A2 + cells in a dose-dependent manner, but not a control cell line HL-60, which was WT1 positive, but HLA-A2 negative (Fig. 1C and D). Furthermore, ESK1 was able to bind primary ovarian cancer cells isolated from a patient ascites (Fig. 1E). Ascites from a HLA-A*02:01 positive patient was stained with mouse ESK1 vs anti-CD45mAb or its isotype control, to distinguish CD45 + leukocytes from cancer cells. Only large tumor cells (CD45−) were positive for ESK1, but not smaller CD45 + lymphocytes. Therefore, ESK1 could selectively recognize ovarian cancer cells in ascitic fluid. ESK1 BiTE also was effective in treating SKOV3 tumors in a xenograft mouse model (Fig. 2). While effector cells alone or the control BiTE showed minimal non-specific killing, the ESK1-BiTE mediated significant tumor inhibition shown by the bioluminescence imaging (Fig. 2A) and total BLI flux (Fig. 2B, C). These data demonstrated the anti-tumor activity of the ESK1-BiTE in vitro and in vivo, which provided the rationale of targeting WT1 in ovarian cancer.

### Design and generation of CAR T-BiTE

**Figure 3:**
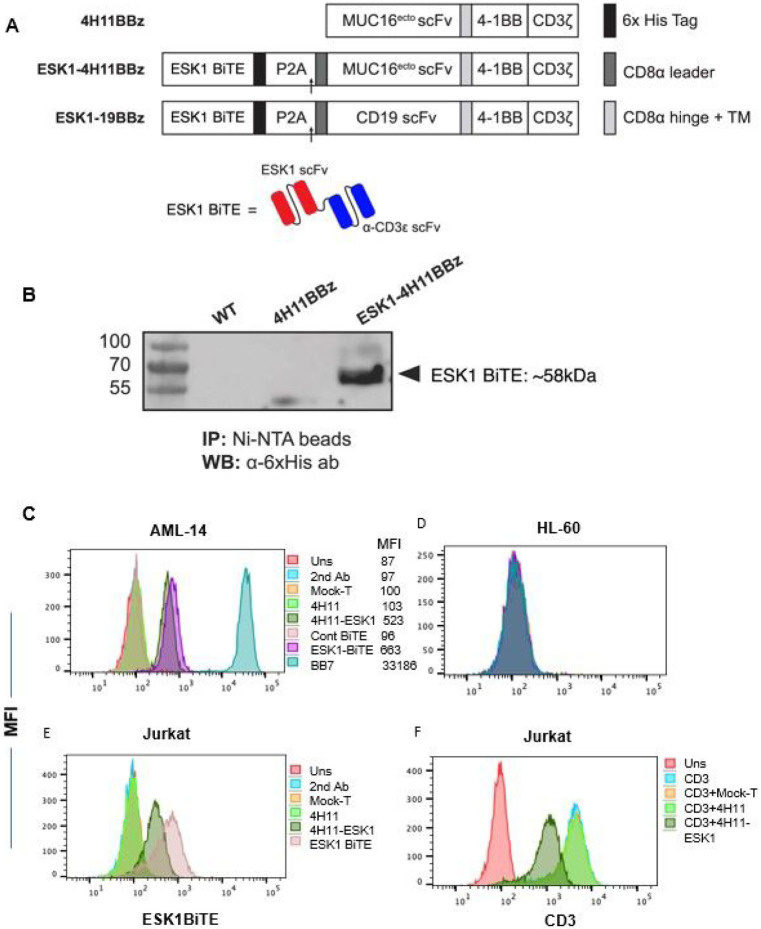
Generation of 4H11 CAR T cells secreting ESK1-BiTE. (A) Schematic of retroviral vectors encoding the 4H11BBz CAR alone (top) and ESK1-4H11BBz CAR constructs encoding a secreted ESK1 bite (bottom). A 6xHis-tag was added at the C-terminal of ESK1 bite for easy detection and affinity purification. A P2A element sequence was used to link ESK1 bite with MUC16 CAR sequences. (B) The (His)6-tag ESK1 bites were pulled down from the medium supernatants of WT, 4H11BBζ, and ESK1-4H11BBζ producer cells using Ni-NTA agarose beads, separated using SDS-PAGE, and analyzed by immunoblotting using an anti-His-tag antibody. The ESK1 bite appeared at the predicted molecular weight (∽58 kDa) in the conditioned media of ESK1-4H11BBζ Galv9 producer cells. (C-E). Supernatant fluids from 4H11, ESK1.BiTE-4H11 or Mock-T cells were concentrated 30-40 fold and used to stain AML-14 (C), HL-60 (D), or Jurkat (E) cells, followed by anti-His tag mAb staining to detect BiTE. Recombinant ESK1 BiTE or its control was used at 10ug/ml. (F). Jurkat cells were blocked by the supernatant fluids from Mock-T, 4H11 or ESK1.BiTE-4H11 CAR T cells (1:1 ratio) on ice for 30 minutes and followed by staining with anti-human CD3 mAb.

ESK1 has potential off target reactivity, possibly limiting its systemic use in humans (29, 30). Therefore, we hypothesized that local delivery of the ESK1 by use of a CAR T cell directed to the tumor might both allow better specificity and also increase efficacy using combined treatment with two orthogonal modalities to prevent heterogeneous tumor cells from escaping. Targeting of two tumor antigens by different modalities could potentially enhance the therapeutic efficacy in heterogenous cancers such as EOC where not all cells highly express a single antigen, such as Muc16 or WT1, at high levels. We therefore engineered CAR-T cells specific for Muc16 to also secrete the ESK1 BiTE. A schematic map of the 4H11 CAR T BiTE vector in comparison to the 4H11 CAR T construct as well as the BiTE structure are shown in Fig. 3A and B. Details are described in the materials and methods.

### ESK1-BiTE is secreted by CAR T cells and binds to targets

We tested if the ESK1-BiTE is secreted by the CAR T cells after transduction. Supernatant fluid from HEK-derived retroviral producer cells (Galv9) demonstrated successful secretion of ESK1 BiTE by western blot at a predicted molecular weight of approximately 55kDa (Fig. 3B). Importantly, secreted BiTE in supernatant from transduced ESK1-4H11 CAR T cells bound specifically to WT1+/HLA-A2 + AML cell line AML-14, but not the HL-60 cell line that is WT1 positive, but HLA-A2 negative (Fig. 3C and D). Thirty-fold concentrated supernatant showed a similar level of binding to AML-14 cell line as 10ug/ml of recombinant ESK1-BiTE used in parallel, suggesting a concentration of about 300ng/ml of BiTE in the conditioned media. This level is well above the EC50 of this BiTE (23). Supernatant fluid from mock-T cells or 4H11 CAR T cells did not bind to AML-14 nor to HL-60, suggesting that the binding by secreted ESK1-BiTE was specific to WT1 RMF/HLA-A2 complex. In addition, supernatant from 4H11-ESK1-BiTE CAR T cells also bound to Jurkat cells (CD3 + T cells) via their anti-CD3 scFv domains, while supernatants from Mock-T cells and 4H11 CART cells did not bind (Fig. 3E). When supernatants were used to block anti-CD3 mAb binding, supernatants from 4H11-ESK1-BiTE CAR T cells partially blocked the anti-CD3 binding, but supernatants from Mock-T or 4H11 CAR T cells did not block binding (Fig. 3F). These results demonstrated that ESK1 was secreted by the CAR T cells at effective levels and has specificity for both WT1 and CD3.

### ESK1-BiTE is secreted by CAR T cells and kills its targets

**Figure 4:**
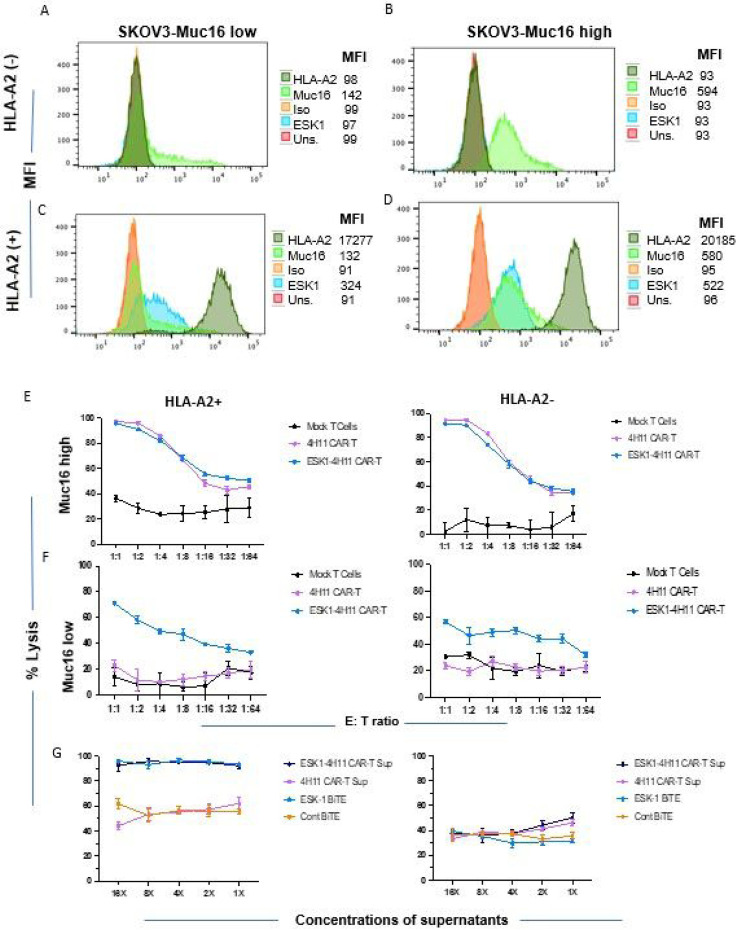
Functional activity of 4H11 CART cells secreting ESK1-BiTE. Expression of Muc16 on SKOV3 wild type (HLA-A2−) or HLA-A2+transduced cells was measured on SKOV3 wild type (Muc16 low) (A and C) or Muc16-transduced cells (Muc16 high) (B and D), using mAb to Muc16. Mouse ESK1 or its isotype control (3ug/ml) was used to measure the expression of WT1 RMF/HLA-A2 complex. (E and F): CAR T cells (10,000/well were serially diluted and incubated for one day to ensure BiTE secretion in the wells. After 1 day of incubation, SKOV3 target cells (10,000 cells/well) and (5,000 Mock T from same donor) were added to all wells for a total of 48 hours of incubation. (G). Supernatants from 4H11 CAR T or ESK1 BITE-secreting CART cells were pooled and concentrated for 16-fold and then serially diluted and incubated with PBMCs as effectors and target SKOV3 cells, at an E:T ratio of 10:1 for 48 hours. ESK1-BiTE and its control BiTE (1ug/ml) was used as control, in a similar serial dilution. The mean shown is the average of triplicate microwell cultures plus minus SD. The data are representative of six separate experiments.

To test if the ESK1-BiTE-secreting 4H11 CAR T cells were able to engage other bystander T cells leading to a greater cytolytic activity than 4H11 CAR T cells alone, we determined the expression of the appropriate antigens (Muc16 and WT1 RMF/HLA-A2 complex) in wild type SKOV3 ovarian cancer cells with “low” Muc 16 expression (Fig. 4A) and those that were transduced with HLA-A2 (Fig. 4C). We also confirmed expression in Muc16 transduced cells with “high” Muc16 expression (Fig. 4B) and those that were also transduced with HLA-A2 (Fig. 4D). CAR T cells were incubated with these target cells and mock-T cells (as bystander effector T cells) from the same donors and the cytotoxicity was evaluated following a 48hr coculture. 4H11 CAR T cells potently killed Muc16 *high* SKOV3 cells, whether A2 + or A2 negative. Consequent to the high Muc16 expression, ESK1-BiTE secretion by these cells did not have additional cytotoxic effects to either of these cell lines (Fig. 4E). In contrast, 4H11 CAR T cells showed no activity above control T cells in killing Muc16 low, SKOV3 cells, whether A2 + or A2 negative (Fig. 4F). However, the ESK1-BiTE-secreting CAR T cells showed significantly greater cytotoxicity against the Muc16 *low* SKOV-3 cell line, than 4H11 CAR T cells alone. Killing was HLA-A2-dependent, and cell dose-dependent. This suggested that secreted ESK1-BiTE could redirect CD3 + T cells to WT1+/HLA-A2 + target cells, thereby enhancing the cytotoxicity by targeting against dual antigens. Low levels of non-dose dependent killing were also seen against the A2 negative target cells, suggesting some non-specific activation of the T cells by the BiTE in these long in vitro cultures (48 hours).

To further investigate if the secreted ESK1 BiTE from 4H11 CART cells was responsible for mediating the T cell cytotoxicity against target cells, the supernatant fluids of 4H11 CAR T or 4H11-ESK1 CAR T cells (concentrated about 16-fold) were incubated with activated T cells (mock-T cells from the same experiments) as effectors along with SKOV3/HLA-A2 positive or negative target cells overnight. Serial diluted supernatant from ESK1-4H11 CART cells lysed the SKOV3/A2 +target cells, but not SKOV3/A2 negative target cells (Fig. 4G). The potency of the secreted ESK1 BiTE cytolytic activity was similar to the control with added recombinant ESK1 BiTE, while control BiTE and Muc16 CAR T cell supernatant fluids were not active and showed similar background killing as the supernatant fluid from 4H11 CAR T cells. Thus, the secreted ESK1 BiTE from the CAR T cells has functional antitumor activity and could account for the enhanced killing activity of the cells shown in the previous experiments. In addition, the BiTE cytotoxic activity did not appear to contribute to the off-target killing.

### Therapy of Muc16 ovarian cancer cells in NSG mice

**Figure 5:**
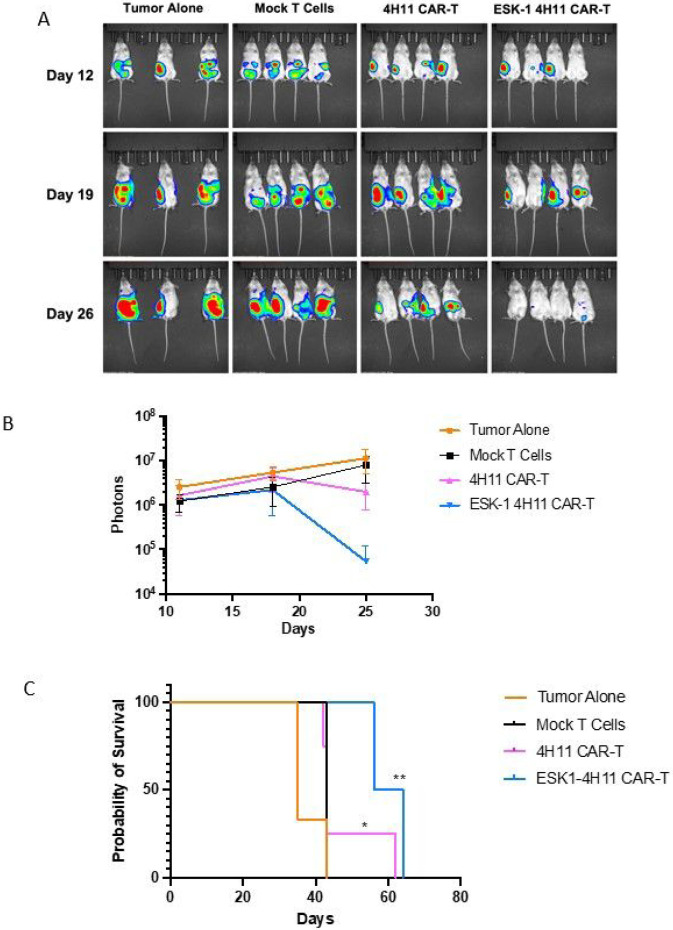
Anti-tumor activity of 4H11 CAR T cells secreting ESK1-BiTE *in vivo*. (A) Muc16 low SKOV3 cells (2 million cells) were injected ip into NSG mice and tumor engraftment was confirmed on day 5 by bioluminescent imaging. 4H11 CAR T cells, ESK1 BiTE-secreting 4H11 CAR T cells, or control mock-T cells (2 million/mouse) were injected ip, followed by injection ip of two million activated T cells (mock-T from the same donor) on the next day. Tumor burden was monitored by bioluminescent imaging on day 12, 19 and 26. (B). Tumor burden of mice in panel A was calculated by summing the luminescent signal of each mouse, and average signal for each group (*n* = 4 per group, except for tumor alone group *n* = 3) plus minus SD, is plotted. Survival of mice from panel A experimental groups was monitored and the significance was evaluated by Mantel-Cox P test. * = <0.05; ** = <0.01 (C).

Based on the in vitro cytotoxicity results, we reasoned that ESK1 BiTE could enhance the anti-tumor activity against Muc16 *low* tumor cells by engaging bystander effector T cells. Therefore, the anti-tumor activity of ESK1-BiTE-secreting 4H11 CAR-T cells was tested *in vivo* using the Muc16 low/HLA-A2 + SKOV3 cell line, in a relevant intraperitoneal (i.p,) model. After confirming the tumor engraftment on day 4 post tumor injection, mice were randomized to different groups. Mock T cells, 4H11 CAR T cells or ESK1 BiTE-secreting 4H11 T cells were injected i.p. into mice. The following day (day 5), mock-T cells from the same donors and the same experiments were injected ip into mice of all groups, as bystander effector T cells, in order to test if ESK1 BiTE was able to redirect these T cells to kill the tumor cells. The tumor growth was monitored by bioluminescence imaging. The enhanced anti-tumor effects of ESK1 BiTE-secreting 4H11 CART cells as compared to the regular 4H11 CAR T cells became evident starting on day 19 and these mice continued to improve. On day 26 post tumor engraftment, there was significant reduction of tumor growth compared to 4H11 CAR T cells and other control groups (Fig. 5A and B). While both 4H11 and ESK1 BiTE-secreting CART cells inhibited tumor growth, the ESK1 BiTE-4H11 CAR T cells had greater anti-tumor activity as compared to the control groups and regular 4H11 CAR T cells (Fig. 5C).

## Discussion

CAR T cell therapy represents an emerging potential treatment option for solid tumors. CAR T cell therapy in EOC has been directed at overexpressed surface antigens, such as HER2, mesothelin, Muc16 and folate receptors. Muc16 can be detected in around 80% of EOC and is expressed to some extent on all the major ovarian subtypes (high grade serous, low grade serous, mucinous, endometrioid, and clear cell), although expression is heterogeneous (31, 32). Because of its up-regulation in tumors and minimal expression on normal tissues, Muc16 is an attractive target in ovarian cancer. Muc16 reactive CAR T cells engineered from 4H11, an antibody that has been extensively tested in human ovarian models and normal tissue with high specificity for tumor cells were studied in a phase I clinical trial(13). Recently, a human bispecific antibody (REGN4018) that binds both Muc16 and CD3, induced T cell activation and killing of Muc16-expressing tumor cells in vitro, even in the presence of soluble CA-125. Furthermore, in a genetically engineered immunocompetent mouse expressing human CD3 and human Muc16 [in humanized target (HuT) mice], REGN4018 inhibited the growth of murine tumors expressing human Muc16. Combination therapy with an anti–PD-1 antibody enhanced its efficacy. This study further validated Muc16 as a potential tumor target for immunotherapy in ovarian cancer (33).

The lack of significant efficacy in a trial of Muc16 CART cells armored with IL12 might be due to the heterogenous or low expression of Muc16 on tumor cells (11, 13). However, in general, CAR T cell therapy in solid tumors including EOC has been far less successful than in hematological cancers (34). In addition to the difficulties caused by T-cell suppression, T cell persistence and possible impaired trafficking to tumor sites, EOC is marked by its extreme heterogeneity within each patient, subtype and metastatic lesion. To date no “ideal” target antigens that are highly and *homogeneously expressed* throughout EOC have been identified. In this study, we chose to create a dual targeting CAR T cell strategy that not only is directed to 2 different antigens, thereby reducing possible escape due to heterogeneity, but also acts to kill by use of two different modalities, that also increases efficacy and reduces possible escape. 4H11 CAR T cells were engineered to secret the ESK1-BiTE, derived from a TCR mimic mAb directed against WT1 epitope presented by HLA-A2, allowing a simultaneous targeting of two antigens that are highly expressed on EOC. Because WT1 is an oncogenic protein, loss of this antigen is also less likely. In addition, the ESK1-BiTE may promote epitope spreading to further extend the killing to a third modality(23). We demonstrated effective secretion of ESK1 BiTE by the 4H11 CART cells and the functional activity of the BiTE both *in vitro* and *in vivo*. While 4H11 CART cells were able to eradicate the SKOV3/HLA-A2 +tumor cells, ESK1-secreting CAR T cells significantly enhanced their anti-tumor activity in Muc16 *low* tumor cells and prolonged the survival of tumor bearing mice.

We selected WT1 RMF presented by HLA-A2 as a model antigen for Muc16 CART cells, because it is a well-validated intracellular tumor antigen found in a wide array of human cancers (20, 35). Targeting of this epitope has been attempted in numerous clinical trials using TCR gene adoptive T cell transfer, or vaccinations in various format against both hematological and solid tumors (19, 20, 22). We previously demonstrated that ESK1-BiTE induces T cell activation and redirects T cell cytotoxicity against primary ovarian cancer cells. In addition to a cytolytic effect on cells presenting WT1 RMF epitope, ESK1-BiTE induced epitope spreading to Her2-derived epitope presented by HLA-A2 molecules, in an autologous system of primary ovarian cancer (23). Here we have further demonstrated therapeutic efficacy of ESK1-BiTE against WT1+/HLA-A2 + SKOV3 ovarian cancer cells in NSG mice. The strategy of targeting both Muc16 and WT1 by use of orthogonal cytolytic modalities offers multiple advantages: 1. Simultaneously targeting two prominent tumor antigens on ovarian cancer cells at once, thereby reducing the risk of antigen loss escape(36); 2. Combining CAR T cells with BiTE into a single gene-modified T cell product improving efficacy by enabling two different modes of killing; 3. Use of locally delivered BiTE to reduce systemic toxicity and possible CRS from excessive T cell activation and off target binding of the BiTE. 4. Increased efficacy following CAR T cells trafficking expansion at the site of the tumor with local concentrations of the ESK1-BiTE, which in turn may recruit, redirect and activate T cells from the tumor microenvironment (TME) and reactivated pre-existing tumor-specific T cells. Indeed, BiTEs have been shown to convert regulatory T cells (T-reg) into cytolytic effector cells (37); thus, this approach could potentially reduce the suppressive tumor microenvironment or convert the suppressive TME to a more immunogenic environment. 5. Improvement of the pharmacokinetics of low molecular weight and rapidly clearing BiTEs by use of continuous secretion at the tumor site. This approach could avoid the need for the continuous infusion typically required by BiTEs. 6. Improving tumor control as the low molecular weight BiTE could penetrate tumor mass more efficiently than CAR T cells; 7. Possible epitope spreading caused by the BiTE engendering additional recruitment of autologous T cells to attack the tumor.

In summary, this study has demonstrated that the targeted delivery by 4H11 CAR T cells, directed to the Muc16 antigen, of the ESK1 BiTE, which reacts to a second, intracellular, tumor antigen, WT1, and which kills by an orthogonal mechanism, could overcome the problem of low Muc16 expression on ovarian cancer cells and therefore is a possible strategy to improve clinical outcome of CAR T cell therapy.

## References

[1] Kroeger PT Jr., and Drapkin R. Pathogenesis and heterogeneity of ovarian cancer. Curr Opin Obstet Gynecol. 2017;29(1):26–34. 27898521 10.1097/GCO.0000000000000340 PMC5201412

[2] Reid BM, Permuth JB, and Sellers TA. Epidemiology of ovarian cancer: a review. Cancer Biol Med. 2017;14(1):9–32. 28443200 10.20892/j.issn.2095-3941.2016.0084 PMC5365187

[3] Cortez AJ, Tudrej R Kujawa KA, and Lisowska KM. Advances in ovarian cancer therapy. Cancer Chemother Pharmacol. 2018;81(1 ):17–38. 29249039 10.1007/s00280-017-3501-8 PMC5754410

[4] Monk BJ, Choi DC, Pugmire G, and Burger RA. Activity of bevacizumab (rhuMAB VEGF) in advanced refractory epithelial ovarian cancer. Gynecol Oncol. 2005;96(3):902–5. 15721449 10.1016/j.ygyno.2004.12.001

[5] Della Pepa C, Tonini G, Pisano C, Di Napoli M, Cecere SC, Tambaro R, Ovarian cancer standard of care: are there real alternatives? Chin J Cancer. 2015;34(1):17–27. 10.5732/cjc.014.10274 PMC4302086 25556615

[6] Sterner RC, and Sterner RM. CAR-T cell therapy: current limitations and potential strategies. Blood Cancer J. 2021;11(4):69. 33824268 10.1038/s41408-021-00459-7 PMC8024391

[7] Maude SL, Frey N, Shaw PA, Aplenc R, Barrett DM, Bunin NJ, Chimeric antigen receptor T cells for sustained remissions in leukemia. N Engl J Med. 2014;371(16):1507–17. 25317870 10.1056/NEJMoa1407222 PMC4267531

[8] Maude SL, Laetsch TW, Buechner J, Rives S, Boyer M, Bittencourt H, Tisagenlecleucel in Children and Young Adults with B-Cell Lymphoblastic Leukemia. N Engl J Med. 2018;378(5):439–48. 29385370 10.1056/NEJMoa1709866 PMC5996391

[9] Rafiq S, Hackett CS, and Brentjens RJ. Engineering strategies to overcome the current roadblocks in CAR T cell therapy. Nat Rev Clin Oncol. 2020;17(3):147–67. 31848460 10.1038/s41571-019-0297-y PMC7223338

[10] Kossaï M, Leary A, Scoazec JY, and Genestie C. Ovarian Cancer: A Heterogeneous Disease. Pathobiology. 2018;85(1-2):41–9. 29020678 10.1159/000479006

[11] Sarivalasis A, Morotti M, Mulvey A, Imbimbo M, and Coukos G. Cell therapies in ovarian cancer. Ther Adv Med Oncol. 2021;13:17588359211008399. 33995591 10.1177/17588359211008399 PMC8072818

[12] Haas AR, Tanyi JL, O'Hara MH, Gladney WL, Lacey SF, Torigian DA, Phase I Study of Lentiviral-Transduced Chimeric Antigen Receptor-Modified T Cells Recognizing Mesothelin in Advanced Solid Cancers. Mol Ther. 2019;27(11):1919–29. 31420241 10.1016/j.ymthe.2019.07.015 PMC6838875

[13] Koneru M, O'Cearbhaill R, Pendharkar S, Spriggs DR, and Brentjens RJ. A phase I clinical trial of adoptive T cell therapy using IL-12 secreting MUC-16(ecto) directed chimeric antigen receptors for recurrent ovarian cancer. J Transl Med. 2015;13:102. 25890361 10.1186/s12967-015-0460-x PMC4438636

[14] Zhang L, Conejo-Garcia JR, Katsaros D, Gimotty PA, Massobrio M, Regnani G, Intratumoral T cells, recurrence, and survival in epithelial ovarian cancer. N Engl J Med. 2003;348(3):203–13. 12529460 10.1056/NEJMoa020177

[15] Sato E, Olson SH, Ahn J, Bundy B, Nishikawa H, Qian F, Intraepithelial CD8+ tumor-infiltrating lymphocytes and a high CD8+/regulatory T cell ratio are associated with favorable prognosis in ovarian cancer. Proc Natl Acad Sci U S A. 2005;102(51):18538–43. 16344461 10.1073/pnas.0509182102 PMC1311741

[16] Goode EL, Block MS, Kalli KR, Vierkant RA, Chen W, Fogarty ZC, Dose-Response Association of CD8+ Tumor-Infiltrating Lymphocytes and Survival Time in High-Grade Serous Ovarian Cancer. JAMA Oncol. 2017;3(12):e173290. 29049607 10.1001/jamaoncol.2017.3290 PMC5744673

[17] Jones HF, Molvi Z, Klatt MG, Dao T, and Scheinberg DA. Empirical and Rational Design of T Cell Receptor-Based Immunotherapies. Front Immunol. 2020;11:585385. 33569049 10.3389/fimmu.2020.585385 PMC7868419

[18] Oka Y, Tsuboi A, Kawakami M, Elisseeva OA, Nakajima H, Udaka K, Development of WT1 peptide cancer vaccine against hematopoietic malignancies and solid cancers. Curr Med Chem. 2006;13(20):2345–52. 16918359 10.2174/092986706777935104

[19] Anguille S, Willemen Y, Lion E, Smits EL, and Berneman ZN. Dendritic cell vaccination in acute myeloid leukemia. Cytotherapy. 2012;14(6):647–56. 22686130 10.3109/14653249.2012.693744

[20] Chapuis AG, Egan DN, Bar M, Schmitt TM, McAfee MS, Paulson KG, T cell receptor gene therapy targeting WT1 prevents acute myeloid leukemia relapse post-transplant. Nat Med. 2019;25(7):1064–72. 31235963 10.1038/s41591-019-0472-9 PMC6982533

[21] Chapuis AG, Ragnarsson GB, Nguyen HN, Chaney CN, Pufnock JS, Schmitt TM, Transferred WT1-reactive CD8+ T cells can mediate antileukemic activity and persist in post-transplant patients. Sci Transl Med. 2013;5(174):174ra27. 10.1126/scitranslmed.3004916 PMC3678970 23447018

[22] Manning-Geist BL, Gnjatic S, Aghajanian C, Konner J, Kim SH, Sarasohn D, Phase I Study of a Multivalent WT1 Peptide Vaccine (Galinpepimut-S) in Combination with Nivolumab in Patients with WT1-Expressing Ovarian Cancer in Second or Third Remission. Cancers (Basel). 2023;15(5). 10.3390/cancers15051458 PMC10001251 36900251

[23] Dao T, Pankov D, Scott A, Korontsvit T, Zakhaleva V, Xu Y, Therapeutic bispecific T-cell engager antibody targeting the intracellular oncoprotein WT1. Nat Biotechnol. 2015;33(10):1079–86. 26389576 10.1038/nbt.3349 PMC4600043

[24] Rafiq S, Purdon TJ, Daniyan AF, Koneru M, Dao T, Liu C, Optimized T-cell receptor-mimic chimeric antigen receptor T cells directed toward the intracellular Wilms Tumor 1 antigen. Leukemia. 2017;31(8):1788–97. 27924074 10.1038/leu.2016.373 PMC5495623

[25] Latouche JB, and Sadelain M. Induction of human cytotoxic T lymphocytes by artificial antigen-presenting cells. Nat Biotechnol. 2000;18(4):405–9. 10748520 10.1038/74455

[26] Brentjens RJ, Davila ML, Riviere I, Park J, Wang X, Cowell LG, CD19-targeted T cells rapidly induce molecular remissions in adults with chemotherapy-refractory acute lymphoblastic leukemia. Sci Transl Med. 2013;5(177):177ra38. 10.1126/scitranslmed.3005930 PMC3742551 23515080

[27] Chekmasova AA, Rao TD, Nikhamin Y, Park KJ, Levine DA, Spriggs DR, Successful eradication of established peritoneal ovarian tumors in SCID-Beige mice following adoptive transfer of T cells genetically targeted to the MUC16 antigen. Clin Cancer Res. 2010;16(14):3594–606. 20628030 10.1158/1078-0432.CCR-10-0192 PMC2907178

[28] Peraro L, Bourne CM, Dacek MM, Akalin E, Park JH, Smith EL, Incorporation of bacterial immunoevasins to protect cell therapies from host antibody-mediated immune rejection. Mol Ther. 2021;29(12):3398–409. 34217891 10.1016/j.ymthe.2021.06.022 PMC8636170

[29] Ataie N, Xiang J, Cheng N, Brea EJ, Lu W, Scheinberg DA, Structure of a TCR-Mimic Antibody with Target Predicts Pharmacogenetics. J Mol Biol. 2016;428(1):194–205. 26688548 10.1016/j.jmb.2015.12.002 PMC4738012

[30] Gejman RS, Jones HF, Klatt MG, Chang AY, Oh CY, Chandran SS, Identification of the Targets of T-cell Receptor Therapeutic Agents and Cells by Use of a High-Throughput Genetic Platform. Cancer Immunol Res. 2020;8(5):672–84. 32184297 10.1158/2326-6066.CIR-19-0745 PMC7310334

[31] Dharma Rao T, Park KJ, Smith-Jones P, Iasonos A, Linkov I, Soslow RA, Novel monoclonal antibodies against the proximal (carboxy-terminal) portions of MUC16. Appl Immunohistochem Mol Morphol. 2010;18(5):462–72. 20453816 10.1097/PAI.0b013e3181dbfcd2 PMC4388147

[32] Koneru M, Purdon TJ, Spriggs D, Koneru S, and Brentjens RJ. IL-12 secreting tumor-targeted chimeric antigen receptor T cells eradicate ovarian tumors in vivo. Oncoimmunology. 2015;4(3):e994446. 25949921 10.4161/2162402X.2014.994446 PMC4404840

[33] Crawford A, Haber L, Kelly MP, Vazzana K, Canova L, Ram R A Mucin 16 bispecific T cell-engaging antibody for the treatment of ovarian cancer. Sci Transl Med. 2019;11(497). 10.1126/scitranslmed.aau7534 31217340

[34] June CH, O'Connor RS, Kawalekar OU, Ghassemi S, and Milone MC. CAR T cell immunotherapy for human cancer. Science. 2018;359(6382):1361–5. 29567707 10.1126/science.aar6711

[35] Sugiyama H. WT1 (Wilms' tumor gene 1): biology and cancer immunotherapy. Jpn J Clin Oncol. 2010;40(5):377–87. 20395243 10.1093/jjco/hyp194

[36] Choi BD, Yu X, Castano AP, Bouffard AA, Schmidts A, Larson RC, CAR-T cells secreting BiTEs circumvent antigen escape without detectable toxicity. Nat Biotechnol. 2019;37(9):1049–58. 31332324 10.1038/s41587-019-0192-1

[37] Choi BD, Gedeon PC, Herndon JE 2nd, Archer GE, Reap EA, Sanchez-Perez L, Human regulatory T cells kill tumor cells through granzyme-dependent cytotoxicity upon retargeting with a bispecific antibody. Cancer Immunol Res. 2013;1(3):163. 24570975 10.1158/2326-6066.CIR-13-0049 PMC3932050

